# Traumatic ulcerative granuloma with stromal eosinophilia mimicking a squamous cell carcinoma

**DOI:** 10.4317/jced.61322

**Published:** 2024-03-01

**Authors:** Natália-Cristina-Trentin Bordignon, Ivan-José Correia-Neto, Rogério Gondak, Ricardo-Luiz-Cavalcanti de Albuquerque-Júnior

**Affiliations:** 1 DDS, MSc, PhD. Department of Pathology, Federal University of Santa Catarina, Postgraduate Program in Dentistry, Florianópolis, Santa Catarina, Brazil; 2 DDS, MSc, PhD student. Piracicaba Dental School, University of Campinas (UNICAMP), Postgraduate Program in Stomatopathology, Piracicaba, São Paulo, Brazil; 3 DDS, MSc, PhD. Department of Pathology, Federal University of Santa Catarina, Health Sciences Center, Florianópolis, Santa Catarina, Brazil

## Abstract

Traumatic ulcerative granuloma with stromal eosinophilia (TUGSE) is a rare lesion of a traumatic-reactive nature of the oral mucosa that can clinically mimic an oral carcinoma. A 59-year-old male patient presented painful ulceration with indurated margins on the base of the tongue, extending to the floor of the mouth. The use of ill-fitting denture hurting the mucosa of the region was reported by the patient. The evolution time was 45 days. The presumptive diagnoses were oral squamous cell carcinoma and chronic ulcer. An incisional biopsy revealed an ulceration associated with an eosinophil-rich inflammatory infiltrate and a bed of proliferating histiocyte-like cells in either diffuse or fasciculate arrangement. There was diffuse immunopositivity for CD3, but focal for CD68 and α-SMA, and negativity for CD30. The final diagnosis was TUGSE. The use of the ill-fitting dental prosthesis was suspended and the lesion had complete spontaneous remission three weeks later. TUGSE is an uncommon traumatic self-limiting lesion that must be included in the differential diagnosis of ulcerative lesions resembling oral cancer. The correlation of clinical and histopathological findings is pivotal for a proper diagnosis, avoiding unnecessary aggressive surgical approaches.

** Key words:**Oral ulcer, eosinophilia, immunohistochemical, differential diagnosis.

## Introduction

Traumatic ulcerative granuloma with stromal eosinophilia (TUGSE) is a rare, reactive, and self-limiting ulcerative lesion restricted to the oral mucosa (1). Clinically, TUGSE manifests as a rapidly increasing ulceration with raised or rolled margins, usually affecting the tongue (2). It can be either asymptomatic or associated with mild to severe pain, and the clinical appearance of the lesion tends to provoke fear of malignancy despite its benign nature, once it may resemble squamous cell carcinoma (3). TUGSE has an uncertain etiology, and although trauma is considered the most frequent cause, the pathological evidence of atypical CD30-positive mononuclear cells within the eosinophil-rich granulation tissue suggests a possible underlying lymphoproliferative disorder (4). Histologically, TUGSE shows a granulation tissue an eosinophil-rich inflammatory infiltrate that typically extends deep into the connective tissue and muscle, and an ulcerated surface composed of fibrin and neutrophils. However, the definite diagnosis should be based on clinical and pathological features, and often supported by a history of trauma (2). TUGSE normally responds favorably to excision of the lesion, with rapid healing of the surgical site, and recurrence in unexpected (5).

In this paper, we report a case of TUGSE in the floor mouth mimicking the clinical appearance of a squamous cell carcinoma. A discussion on the clinicopathologic features and differential diagnosis of the lesion, as well as a brief review of the literature addressing this debate, are also provided.

## Case Report

**Figure 1:**
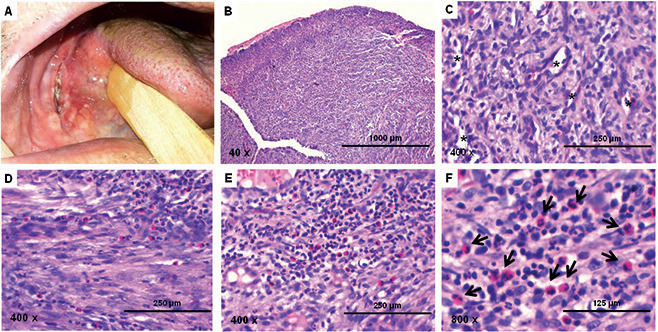
(A) Clinical examination showing an ulceration with and slightly raised bright-red borders and bed covered by white-yellow fibrinous pseudomembrane. (B) HE-stained histological slides showing the oral ulcerated oral mucosa and granulation tissue underlying (40 x). (C) Details of the granulation tissue rich in capillary vessels associated with intense inflammatory infiltrate (400 x). (D) Fasciculated proliferation of histiocyte-like cells permeated by eosinophils (400 x). (E) Intense inflammatory infiltrate composed of lymphocyte, neutrophils and eosinophils (400 x). (E) Detail of the eosinophils (800 x). Captions: asterisks – capillary vessels; arrows: eosinophils.

**Figure 2:**
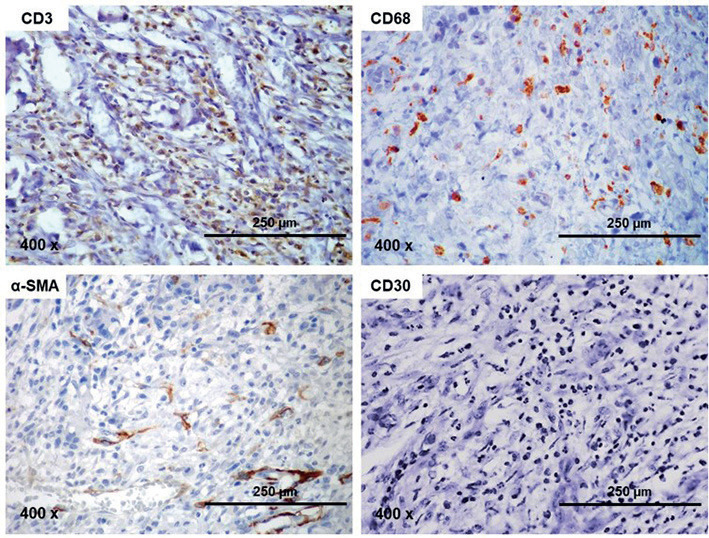
Immunohistochemical analysis of the lesion. (A) Diffuse positivity for CD3 in lymphocytes and (B) focal and sparse positivity for CD68 in histiocyte-like cells. (C) Positivity for α-SMA was mainly observed surrounding small vessels, and focally in some stromal spindle cells. (D) CD30 showed to be negative (SABC, 400 x).

A 59-year-old Caucasian man, rural worker, was referred to a private dentistry service with chief complaint of a painful wound in the mouth that had been noticed approximately 1.5 months ago. The patient’s medical history included controlled hypertension managed with oral administration of Captopril 50 mg. The patient revealed a history of chronic smoking and occasional use of alcohol, as well as a family history of diabetes. Extra oral examination revealed no lymphadenopathy or face swelling, but a marked loss of vertical dimension of occlusion was observed. Intraoral examination revealed lower total edentulism and the presence of a 3.5 cm indurated ulceration covered by white-yellow fibrinous pseudomembrane, with slightly raised bright-red borders. The lesion was located on the right side of the base of the tongue extending to the floor of the mouth (Fig. 1A). The patient also reported the use of ill-fitting denture hurting the mucosa of the region. Based on the clinical findings, the presumptive diagnoses of squamous cell carcinoma and traumatic chronic ulcer were established and an incisional biopsy was performed. Pathological analysis of the sample showed ulcerated oral mucosa covered by fibrinous exudate and polymorphonuclear infiltrate. Adjacent, there was exuberant granulation tissue, forming a dense network of small immature capillary vessels, permeated by an intense infiltration of lymphocytes, macrophages and neutrophils, together with a large number of eosinophils. In addition, a diffuse and sometimes fasciculated proliferation of fusiform, ovoid and histiocytoid cells was observed amid the granulation tissue, particularly at the bottom of the surgical specimen (Fig. 1 B-F). Immunohistochemical analysis showed diffuse positivity for CD3, but focal and dispersed for CD68, whereas positivity for α-SMA was limited to the periphery of capillary vessels sparse stromal cells and CD30 was negative (Fig. 2). Based on clinical, pathological and immunohistochemical features, the final diagnosis was TUGSE. The use of the traumatic prosthetic device was suspended and the patient was subjected to weekly clinical follow-up. Approximately 3 weeks later, complete spontaneous remission of the ulcer was observed. The patient was also advised to seek new prosthetic dental rehabilitation.

## Discussion

TUGSE is a relatively rare lesion of the oral mucosa whose etiology and pathogenesis remain uncertain. It commonly involves the tongue surfaces but can occasionally be found in other oral Anatomic sites, such as buccal mucosa, floor mouth, retromolar area and lips (6). When TUGSE affects nursing infants, often on the anterior ventral surface of the tongue, it is frequently called Riga-Fede disease (7,8). We found 38 well-documented cases of TUGSE in the oral tissues between 2010 and 2022, including the current case (Table 1,  Table 1 cont.). The mean age of the patients was 55.5 ± 20.3 years, and the median was 59 years. Most of the patients were above 40 years-old (76.3%). The most affected anatomic site was the tongue (56.6%) and pain was reported in 44.73% of the cases. All those data were in accordance with those previously described by Shen *et al*. in a series of 34 cases of TUGSE reported between 2003 and 2009 (6). However, unlike the predominance of females observed by those authors, we found no sex predilection d in the current series, although the determinants of this supposed change in this demographic parameter are not entirely clear. Clinical features of TUGSE, such as rapid expansion and ulceration with typically indurated and rolled-appearing margins, just as observed in the current case, often lead to a presumptive misdiagnosis of malignancy despite its benign nature (3,5). Therefore, performing an incisional biopsy and histopathological analysis may be pivotal for establishing a correct diagnosis and, consequently, an appropriate therapeutic approach.

**Table 1:**
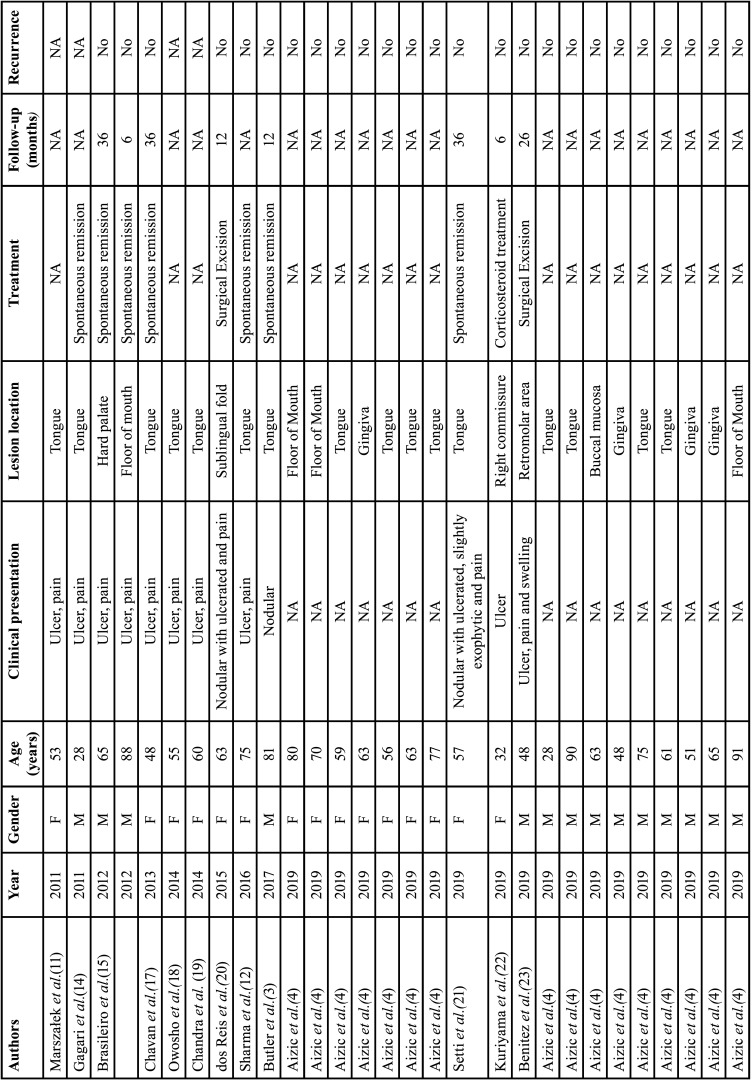
Demographic and clinicopathological data of 38 patients with traumatic ulcerative granuloma with stromal eosinophilia (TUGSE).

**Table 1 cont.**
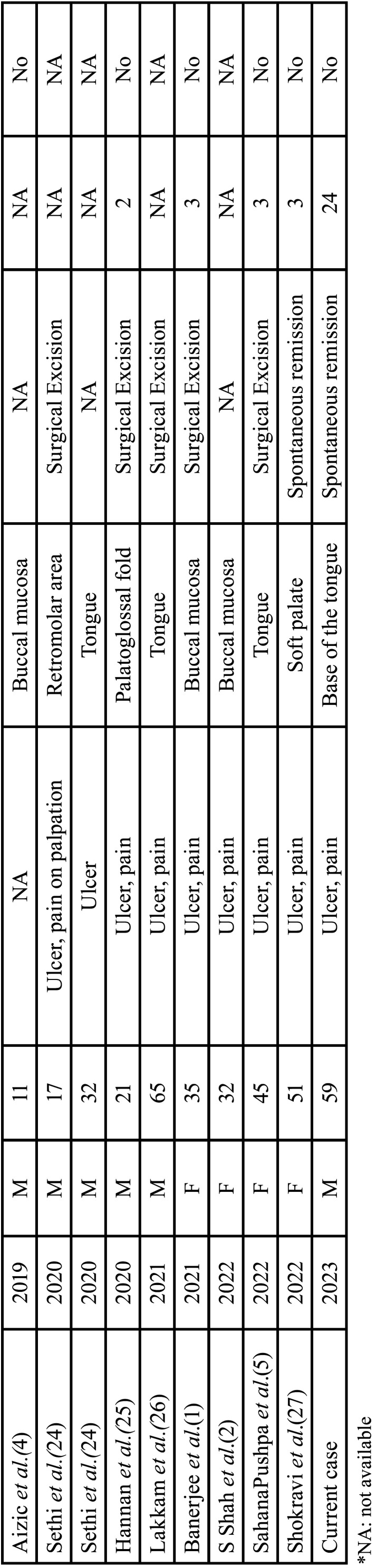
Demographic and clinicopathological data of 38 patients with traumatic ulcerative granuloma with stromal eosinophilia (TUGSE).

Histologically, TUGSE is typically characterized by granulation tissue associated with inflammatory infiltrate rich in eosinophils. In some cases, the eosinophils are preferentially located around degenerated muscle fibers in the bottom of the lesion, suggesting a possible relation with muscular injury (5,9). Although most of these features were found in the current case, the eosinophils showed a diffuse distribution in the lesion, as also reported by Shen *et al*. (6). Hence, the potential relation with muscle degenerative changes could not be attested in the current case. Large atypical mononuclear cells with both lymphocyte and histiocyte morphological pattern have been reported in some cases of TUGSE (10). This leads to some concern that TUGSE may represent an oral counterpart of cutaneous lymphomatoid papulosis, with a potential for transformation into lymphoma, and consecutively to recommendations for margin-free excision and long-term follow-up in TUGSE with atypical cells. Moreover, those atypical cells that are positive for T-cell markers, including CD30, have been reported in TUGSE (6). As CD 30-positive cells have been associated with lymphomas, such as mycosis fungoides, T cell lymphoma and anaplastic large cell lymphoma, as well as with borderline CD30+ lesions, some authors have speculated about the possibility that TUGSE represents a lymphoproliferative disorder (3). However, in the series of 17 cases reported by Aizic *et al*. in 2019, only seven (41.17%) were positive for CD30, and none of them were monoclonal for TCR, ruling out the possibility of a potentially malignant nature (4). In the current case, the positivity of CD3, a T cell marker, limited to typical inflammatory lymphocytes, but negativity for CD30, supports the theory that TUGSE is a benign reactive lesion rather than a potentially malignant disorder. Although TUGSE is typically composed of larger histiocyte-like cells, their precise nature is not fully clarified, but a possible histiocytic and myofibroblastic origin has been proposed (11). Although large histiocyte cells were abundant in the histological slices of the current case, frankly atypical mononuclear cells were not found. Furthermore, as positivity for CD68, a histiocytic differentiation marker, and α-SMA, a myofibroblastic marker, were focal, it is more likely that histiocytes and myofibroblasts are just satellite cells.

Most cases of TUGSE appear to undergo spontaneous remission spontaneously resolves on its own, as occurred in the current case, and symptomatic treatment only is the mainstay of therapy. Once trauma is the major etiological factor of this injury, the patient should be referred to a dentist to treat the underlying causes, such as potentially traumatic parafunctional habits and prosthetic, orthodontics or other dental devices (3). Hence, these measures were also adopted in the present case. Other reasonable conservative modalities of management to improve healing time in an otherwise healthy individual include the topical use of a 0.1% triamcinolone acetonide mouthwash, electrocoagulation, and liquid nitrogen (5). However, although surgical excision is also an accepTable form of treatment for TUGSE, providing rapid healing after excision of the lesion. Special attention needs to be given to the histopathological diagnosis of this lesion because it may present clinical features that mimic malignancy, leading inadvertently to an overtreatment (12).

Recurrence is not expected in cases of TUGSE. In the series of 34 cases reported by Shen *et al*. in 2015, relapse of the lesion occurred in only one case, likely due to failure to eliminate the causative factors such as sharp tooth margins and ill-fitting prostheses (6). In addition, recurrence was not reported in any of the cases reported herein, even though no information about this issue was provided in eight cases (21;05%). However, despite the favorable prognosis presented by TUGSE, a minimum of two years of follow-up is recommended by Sarangarajan *et al*., particularly to ensure that the persistence of parafunctional habits or other traumatic factors can determine the recurrence of the lesion (13).

In conclusion, TUGSE is an uncommon self-limiting lesion of a traumatic nature whose clinical appearance can mimic oral squamous cell carcinoma. The diagnosis is based on the correlation of those clinical aspects with histopathological features of the lesion, mainly the ulceration infiltrated by inflammatory cells, predominantly eosinophils. Therefore, TUGSE must be included in the differential diagnosis for ulcers that have a close resemblance to squamous cell carcinoma. In addition, physicians and dental professionals must be aware of the diagnostic, therapeutic, and prognostic implications of TUGSE to avoid unnecessary aggressive therapeutic approaches.
